# Gesture Use and Processing: A Review on Individual Differences in Cognitive Resources

**DOI:** 10.3389/fpsyg.2020.573555

**Published:** 2020-11-05

**Authors:** Demet Özer, Tilbe Göksun

**Affiliations:** Department of Psychology, Koç University, Istanbul, Turkey

**Keywords:** individual differences, gesture production, gesture processing, cognitive resources, functions of gestures

## Abstract

Speakers use spontaneous hand gestures as they speak and think. These gestures serve many functions for speakers who produce them as well as for listeners who observe them. To date, studies in the gesture literature mostly focused on group-comparisons or the external sources of variation to examine when people use, process, and benefit from using and observing gestures. However, there are also internal sources of variation in gesture use and processing. People differ in how frequently they use gestures, how salient their gestures are, for what purposes they produce gestures, and how much they benefit from using and seeing gestures during comprehension and learning depending on their cognitive dispositions. This review addresses how individual differences in different cognitive skills relate to how people employ gestures in production and comprehension across different ages (from infancy through adulthood to healthy aging) from a functionalist perspective. We conclude that speakers and listeners can use gestures as a compensation tool during communication and thinking that interacts with individuals’ cognitive dispositions.

## Introduction

Human language occurs in a face-to-face interactional setting with the exchange of multiple multimodal cues such as eye-gaze, lip movements, body posture, and hand gestures. In this review paper, we focus on one of these multimodal cues: iconic hand gestures (henceforth, gestures) that represent objects, events, and actions. Speakers use an abundant number of gestures as they speak or think. These gestures serve many functions for speakers who produce them and for listeners who observe them (Goldin-Meadow et al., 2001; McNeill, 2005; Özyürek, 2014; Kita et al., 2017; Novack and Goldin-Meadow, 2017; Dargue et al., 2019). Although gesture and speech express meaning in a coordinated and integrated manner, gesturing is not mandatory for communication and, hence, shows variation across situations and individuals (Kita and Özyürek, 2003; Kendon, 2004; McNeill, 2005; Streeck, 2009). Speakers differ in how frequently they use gestures, how salient their gestures are, and how much they benefit from *using* gestures during encoding and learning. On the other hand, listeners also differ in how much they attend to the speaker’s gestures and benefit from *observing* gestures during comprehension and learning. The current paper will discuss individual differences in gesture use and processing.

There is individual variation in all human traits. People exhibit individual differences in cognitive abilities such as working memory (WM) capacity, attention, speech production, and processing as well as language acquisition (e.g., Daneman and Green, 1986; Just and Carpenter, 1992; Bates et al., 1995; Kane and Engle, 2002; Broadway and Engle, 2011; Huettig and Janse, 2016; Kidd et al., 2018). Current theories in cognitive science have not fully accounted for the existence as well as the causes of these individual differences for scientific gain (Underwood, 1975; Vogel and Awh, 2008). Most of the earlier studies in the gesture literature disregarded the variation among individuals and focused on group comparisons based on age (e.g., Feyereisen and Havard, 1999; Colletta et al., 2010; Austin and Sweller, 2014; Özer et al., 2017), sex (e.g., Özçalışkan and Goldin-Meadow, 2010), neuropsychological impairments (e.g., Cleary et al., 2011; Göksun et al., 2013b, 2015; Akbıyık et al., 2018; Akhavan et al., 2018; Hilverman et al., 2018; Özer et al., 2019; see Clough and Duff, 2020 for a review), culture, and the native status of the speakers and the listeners (i.e., bilinguals vs. monolinguals; e.g., Goldin-Meadow and Saltzman, 2000; Mayberry and Nicoladis, 2000; Pika et al., 2006; Kita, 2009; Nicoladis et al., 2009; Gullberg, 2010; Smithson et al., 2011; Kim and Lausberg, 2018; Azar et al., 2019, 2020) to understand how human multimodal language faculty operates at a general level. The gesture theories and current experimental practices in the gesture literature mostly downplayed the significance of individual differences and treated them as error variance. These studies create an illusionary and incorrect assumption that gesturing and the cognitive and communicative benefits of using and seeing gestures are invariant across people. However, using and observing gestures show not only across-group but also within-group variation (e.g., Hostetter and Alibali, 2007, 2011; Chu et al., 2014; Wu and Coulson, 2014a,b; Dargue et al., 2019; Özer et al., 2019; Özer and Göksun, 2020). What drives this variation?

There are external and internal sources of variation in gesture use and processing. The external sources of variation could be speech content (e.g., spatial vs. non-spatial topics; Rauscher et al., 1996; Feyereisen and Havard, 1999; Lausberg and Kita, 2003; Alibali, 2005; Hostetter, 2011), communicative context (e.g., the visibility between interlocutors, communicative intention, and audience design; Alibali et al., 2001; Trujillo et al., 2018; Schubotz et al., 2019), task difficulty and cognitive load levels (e.g., complex spatial tasks such as mental rotation; Wesp et al., 2001; Kita and Davies, 2009). There are also internal sources variation; even under the same external circumstances, people can behave differently. Insights into which mechanisms contribute to these individual differences just started to emerge (e.g., Hostetter and Alibali, 2007, 2011; Chu et al., 2014; Wu and Coulson, 2014a,b; Dargue et al., 2019; Aldugom et al., 2020; Kartalkanat and Göksun, 2020; Özer and Göksun, 2020).

Individual differences in personality characteristics, age, cognitive, and perceptual skills contribute to variation among individuals in terms of gesture use and processing (e.g., Vanetti and Allen, 1988; Cohen and Borsoi, 1996; Hostetter and Alibali, 2007, 2011; Wartenburger et al., 2010; Hostetter and Potthoff, 2012; Marstaller and Burianová, 2013; Göksun et al., 2013a; Chu et al., 2014; Gillespie et al., 2014; Wu and Coulson, 2014a,b; Pouw et al., 2016; Austin and Sweller, 2017, 2018; Eielts et al., 2018; Galati et al., 2018; Dargue and Sweller, 2020; Kartalkanat and Göksun, 2020; Özer and Göksun, 2020). However, most of the research on individual differences focused on gesture production, particularly on the cognitive correlates of variation in spontaneous gesture use and how much people benefit from using gestures during problem-solving and encoding of information. Research on individual variation in how listeners attend to speakers’ gestures and benefit from observing gestures for comprehension and learning is limited (Wu and Coulson, 2014a,b; Aldugom et al., 2020; Özer and Göksun, 2020).

In the current review paper, we discuss individual differences in (1) *gesture use*: how frequently speakers use gestures during spontaneous speech and how much they benefit from using gestures during task solving and learning and (2) *gesture processing*: how listeners attend to and process speakers’ gestures and how much they benefit from observing speakers’ gestures for online comprehension or subsequent learning. We specifically focus on individual differences in cognitive and perceptual abilities (see Hostetter and Potthoff, 2012 for personality characteristics). This review has three highlights: (1) we attempt to bring a complete picture of individual differences in gesture by bridging production (i.e., using gestures) and comprehension (e.g., seeing gestures) fields. (2) We adopt a *functionalist* approach to discuss possible cognitive correlates of gesture use and processing. *Functionalist gesture theories* (as opposed to *mechanistic approaches* such as McNeill, 1992, 2005; Hostetter and Alibali, 2008) discuss *why* speakers use gestures and what *functions* gestures serve for speakers and listeners during communication and thinking (e.g., Kita and Özyürek, 2003; Pouw et al., 2014; Cook and Fenn, 2017; Kita et al., 2017; Novack and Goldin-Meadow, 2017). Theories asserting for what purposes speakers and listeners employ gestures might inform us about the possible cognitive correlates of individual differences in gesture use and processing. (3) We also take on a life-span developmental perspective, which covers how gesture use and processing differ in changing cognitive skills throughout the developmental trajectory (from childhood through adulthood to healthy aging).

The literature on how different populations across ages use and process gestures during communication and learning is quite rich. It is noteworthy that the current paper is not a comprehensive review of the general literature. Instead, we specifically focus on studies investigating individual differences in these processes. We first review the functions of gestures during communication and learning (section Functions of Gestures During Communication and Learning). Then, we address evidence on individual differences in gesture use (section Individual Differences in Gesture Production) and gesture processing (section Individual Differences in Gesture Processing) for children, young adults, and elderly adults. Last, we conclude the current state of the field and discuss areas that are open to further investigation (section Conclusion and Future Directions).

## Functions of Gestures During Communication and Learning

Several theories suggest how and why gestures occur during communication and thinking. Mechanistic theories mostly propose *how* gestures arise during communication and thinking (e.g., McNeill, 1992, 2005; Hostetter and Alibali, 2008, 2018). Functionalist theories, on the other hand, try to explain *why* we use gestures and the functions that gestures serve during communication and thinking, both for the speaker and the listener (e.g., Goldin-Meadow et al., 2001; Kita and Özyürek, 2003; Pouw et al., 2014; Cook and Fenn, 2017; Kita et al., 2017; Novack and Goldin-Meadow, 2017). The approach in this review will be from functionalist perspectives as they can give insight into which mechanisms might contribute to individual differences in gesture use and processing.

Gestures have several functions during communication and thinking. First, gestures affect communication between interlocutors. Speakers and listeners employ gestures for communicative purposes. Speakers produce gestures to communicate information, and listeners, in turn, benefit from these gestures to comprehend the to-be-communicated message (e.g., Beattie and Shovelton, 1999; Alibali et al., 2001; Holler and Stevens, 2007; Hostetter, 2011; Goldin-Meadow and Alibali, 2013). Speakers use gestures as an alternative channel of expression. Hence, both speakers and listeners employ gestures more in communicative challenges stemming from cognitive dispositions such as when a speaker is linguistically non-competent (e.g., bilinguals talking in their non-native language; Smithson et al., 2011; Gullberg, 2010) or has hearing impairments (Obermeier et al., 2012). The communicative function of gestures suggests that speakers and listeners with low communicative capacity (e.g., low linguistic proficiency, low semantic fluency, or the non-native status of the speaker and the listener) might employ and benefit from gestures more.

Second, gestures affect speakers’ and listeners’ cognitive processes. Gestures help activate, maintain, manipulate, and package visual, spatial, and motoric information for speaking and thinking (Kita et al., 2017). Gestures reduce cognitive load by keeping spatial-motoric information active in WM (Goldin-Meadow et al., 2001; Wesp et al., 2001; Morsella and Krauss, 2004; Ping and Goldin-Meadow, 2010; Cook et al., 2012; Marstaller and Burianová, 2013) and by projecting internal representations to an external space (e.g., Pouw et al., 2014). Producing gestures provides an external visual feedback that can be used to maintain or retrieve task-related visual-spatial information and, hence, reduces the cognitive load. Considering this, we expect that people might use gestures as a compensatory tool to manage their cognitive load. For example, people with lower visual-spatial cognitive capacity (e.g., lower visual-spatial WM capacity, lower general spatial skills assessed by mental rotation, and lower fluid intelligence assessed by Raven’s Matrices) might use gestures more frequently to compensate for their limited resources when talking and thinking, especially about spatial information (e.g., Trafton et al., 2006; Göksun et al., 2013a; Chu et al., 2014; Galati et al., 2018). In a similar vein, speakers’ gestures provide a stable visual representation for observers (i.e., listeners) and help listeners during comprehension and learning. People with lower cognitive resources might be in a greater need for external aids, and thus benefit more from seeing gestures (e.g., de Nooijer et al., 2013; Wu and Coulson, 2014a; Özer and Göksun, 2020).

Functional gesture theories assert that gestures help to convey information during communication and manage cognitive load during speaking, thinking, and learning (e.g., Kita et al., 2017; Novack and Goldin-Meadow, 2017). This suggests that gesture use and processing are sensitive to the cognitive dispositions of the speakers and the listeners. People might convey gestures to manage and compensate for their limited cognitive resources.

Mechanistic gesture theories, on the other hand, emphasize how people employ gestures. As opposed to functionalist theories, one of the first and most influential mechanistic accounts of gesture production (*The Growth Point Theory*, McNeill, 1992, 2005; McNeill and Duncan, 2000) posits that gestures do not compensate for thinking and speaking. According to this account, gesture and speech originate from a single representational system, where an utterance contains both linguistic and imagistic structures that cannot be separated. Speech stems from propositional linguistic representations and gestures stem from non-propositional imagistic representations and reflect visual, spatial, and motoric thinking (McNeill, 1992, 2005; Krauss et al., 2000). This account suggests that gestures are the manifestations of the imagistic component of the thought. Although mechanistic accounts would not be against the role gestures play for people to manage cognitive processes, they emphasize *how* people employ gestures rather than *why* they gesture.

In the following sections, we review evidence regarding how individual differences in cognitive domains relate with gesture use and processing from a functionalist account, mainly considering the *gesture-as-a-compensation-tool* view. That is, following the functionalist approach, we will illustrate the functions of gestures for speakers and listeners who use their cognitive resources differently. Gestures might not be used as a compensatory tool for every situation across different groups (e.g., So et al., 2009; Chui, 2011; de Ruiter et al., 2012); yet, the current state of the field supports the beneficial part of gestures for communication, thinking, and learning (e.g., Goldin-Meadow et al., 2001; Kita et al., 2017; Novack and Goldin-Meadow, 2017).

## Individual Differences in Gesture Production

People from all ages show variation in terms of how frequently they use gestures, how salient their gestures are, and what types of gestures they use during spontaneous speech (e.g., Feyereisen and Havard, 1999; Richmond et al., 2003; Priesters and Mittelberg, 2013; Chu et al., 2014; Nagels et al., 2015; Schmalenbach et al., 2017; Arslan and Göksun, in press). People also differ in how much they benefit from using gestures during speaking, encoding, and subsequent learning (e.g., Goldin-Meadow et al., 2001; Ping and Goldin-Meadow, 2010; Galati et al., 2018). To date, studies mostly focused on two possible cognitive correlates: visual-spatial vs. verbal cognitive resources. We discuss how individual differences in visual-spatial and verbal cognitive capacities relate to gesture production in children, adults, and elderly adults.

### Individual Differences in Gesture Production in Children

Babies start to use pointing gestures at around 12 months of age and iconic gestures at around 3 years of age (Iverson et al., 1999; Özçalışkan and Goldin-Meadow, 2005, 2010). Gestures open the way for the transition from prelinguistic to linguistic period, and gestures become increasingly intertwined with speech as children become older (e.g., Capirci et al., 2005; Capirci and Volterra, 2008; Liszkowski et al., 2008). Özçalışkan and Goldin-Meadow (2005) analyzed children’s gestures at 14, 18, and 22 months of ages when children are interacting spontaneously with their mothers. They showed that children used more gestures as they got older. Moreover, there was a developmental shift toward the use of more supplementary gestures (e.g., saying “ride” and pointing at the bike) as opposed to reinforcing gestures (e.g., saying “bike” and pointing at the bike) by older children. Yet, there was no difference in the quality or the quantity of the maternal input across development, suggesting that changes in children’s gestural behavior might reflect developmental changes in children’s own cognitive processes. Then, individual differences in several cognitive processes might lead to variations in how and to what extent children use gestures in spontaneous speech. Children, even as early as 14 months of age, show individual variation in whether they use iconic gestures and how frequently they use them (e.g., Iverson et al., 1999; Özçalışkan and Goldin-Meadow, 2005). What drives these very early individual differences in gesture use? To date, the gesturing behavior of young children mostly focused on how individual differences in early gesture use predicted later language development (e.g., Rowe and Goldin-Meadow, 2009; Demir et al., 2015). Studies examining the precursors of these variations, on the other hand, primarily focused on how parental language input (speech and gesture) relates with children’s spontaneous gesture production (e.g., Iverson et al., 2008; Rowe et al., 2008; Tamis-LeMonda et al., 2012). It is unknown which cognitive and perceptual abilities of these young children drive early individual differences in gesture production. Early socio-cognitive precursors of gesture use in infancy is an open area for further investigation.

How do children use gestures at later ages, such as during preschool and school-age? Children have not yet fully developed verbal skills as compared to young adults; thus, they might use gestures more during speaking as gestures provide an alternative channel of expression (e.g., Melinger and Levelt, 2004) and help facilitate speaking (Krauss et al., 2000). Indeed, studies report that preschool-aged children benefit more from gestures than older children and adults, especially when using complex language (e.g., Church et al., 2000; Austin and Sweller, 2014). Moreover, children in transitional stages (i.e., children who have the conceptual knowledge but not yet the skills to verbalize that knowledge) used more gestures to convey ideas compared to children who had necessary verbal resources to convey the same idea linguistically (e.g., Church and Goldin-Meadow, 1986; Perry et al., 1992). These gestures (so-called *gesture mismatches*) expressed non-redundant information that was not found in the accompanying speech. Children (ages 5–10) used more non-redundant speech-gesture combinations both at the clause and word levels compared to adults (Alibali et al., 2009). This is also evident in the expression of other linguistically challenging categories such as causal or spatial relations (e.g., Göksun et al., 2010; Austin and Sweller, 2018; Calero et al., 2019; Karadöller et al., 2019). Children used more gestures to convey additional information when they could not verbalize instruments of causal events (Göksun et al., 2010) or spatial relations such as left-right (Karadöller et al., 2019). For example, ambiguous spatial terms such as “*here*” can be complemented by gestures to specify the spatial relation (Karadöller et al., 2019). The multimodal discourse continues to develop during the school-age years. There is a developmental shift toward the use of a higher number of gestures per clause by 10-year-old children and adults than 6-year olds in narrative production tasks (e.g., Colletta et al., 2010; Alamillo et al., 2013).

Developmental studies suggest that children might use gestures as an alternative channel of expression to compensate for their limited linguistic proficiency (e.g., younger vs. older children or children vs. adults; Church et al., 2000; Alibali et al., 2009; Colletta et al., 2010). This is in line with bilingualism research showing that bilingual children speaking in their L2 used more gestures than monolinguals (e.g., Smithson et al., 2011; Wermelinger et al., 2020). Moreover, research on clinical populations with communication and language delays suggests that although there are delays in gesture production in the first 2 years, gesture might be used to compensate for communication and language difficulties at preschool and school ages by some children (Özçalışkan et al., 2013; LeBarton and Iverson, 2017). Children with language impairments (LI) used gestures at a higher rate and produced greater proportions of gestures that added unique information to the accompanying speech compared to typically developing (TD) peers, suggesting that children with LI employ gestures as an alternative channel of expression in the face of language difficulties (Evans et al., 2001; Blake et al., 2008; Iverson and Braddock, 2011; Mainela-Arnold et al., 2011, 2014).

Similar to children with LI, children with Down syndrome (DS) used more gesture-only expressions and expressed information uniquely in their gestures compared to TD children to compensate for spoken language delays (Stefanini et al., 2007; Dimitrova et al., 2016; Özçalışkan et al., 2017). Children with Williams syndrome (WS) also used more iconic gestures in a picture naming task compared to TD children to alleviate their word-finding difficulties (Bello et al., 2004). Yet, not all children with language delays benefit from gestures as a compensatory tool. Children with autism spectrum disorder (ASD) exhibit delays in gesture production that are apparent both in frequency and complexity (Colgan et al., 2006; Rozga et al., 2011; Watson et al., 2013; Dimitrova et al., 2016; Özçalışkan et al., 2016, 2017). Research shows that children with ASD used gestures to initiate and sustain joint attention and to compensate for speech limitations by supplementing speech to a lesser degree compared to TD peers, leading to negative consequences for learning and social interaction opportunities (Sowden et al., 2013; Watson et al., 2013; Mastrogiuseppe et al., 2015). Impairments in gesture production are more pronounced in ASD compared to other developmental delays such as DS (Mastrogiuseppe et al., 2015), LI (Stone et al., 1997), and general intellectual delay (Mundy et al., 1990) and, thus, are considered to be a central component of problems in social interactions and delays in social development in ASD. Moreover, language delays not only affect children’s gesture productions but also gestural input they receive from their caregivers, resulting in cascading consequences for language development.

Research suggests that children’s language level affects caregivers’ gestures to a greater extent when a child’s language skills are limited (Iverson et al., 2006; Talbott et al., 2015; Dimitrova et al., 2016; Özçalışkan et al., 2017, 2018). For example, mothers of non-diagnosed high-risk ASD infants gestured more frequently compared to mothers of low-risk ASD infants (Talbott et al., 2015). The evidence on the compensatory use of gestures by children with language delays indicate that gesture is a tool that should be harnessed to support learning, especially for child clinical populations (LeBarton and Iverson, 2017). Gesture is also an early diagnostic tool to foresee persistent language delay, especially for children with unilateral brain lesions (Sauer et al., 2010; Özçalışkan et al., 2013). Although these studies suggest a link between early spoken language abilities and gesture production in children, the direct evidence on how individual differences in early receptive and expressive language skills relate with spontaneous gesture use within children with and without language delays is quite limited (Kartalkanat and Göksun, 2020; Wermelinger et al., 2020).

A growing body of literature shows that using gestures benefit children’s subsequent memory and learning (e.g., Alibali and DiRusso, 1999; Wakefield et al., 2018). Do all children benefit similarly from using gestures? Post et al. (2013) showed that children who simultaneously produced and observed gestures when learning grammatical rules performed worse than children who only observed gestures. However, the adverse effects of gesturing on learning were only visible for children with lower verbal skills, suggesting that producing and observing gestures simultaneously might be too cognitively demanding, especially for children with lower verbal resources (Kalyuga, 2007). Nevertheless, it should be noted that this study tested the effects of using gestures on learning under high cognitive load. There is no direct evidence on how verbal skills relate to how much children benefit from using gestures when they are under average cognitive load (e.g., without observing gestures simultaneously).

Developmental studies mostly compared different age groups (e.g., children vs. adults or younger vs. older children; e.g., Colletta et al., 2010), bilinguals vs. monolinguals (e.g., Mayberry and Nicoladis, 2000; Smithson et al., 2011), and clinical vs. non-clinical groups (e.g., Bello et al., 2004; Dimitrova et al., 2016; LeBarton and Iverson, 2017). These studies suggest that children use gestures as a compensatory tool, and individual differences in verbal skills play a role in how much children use and benefit from using gestures during learning. Moreover, visual-spatial skills follow a protracted development, and children show individual variation in visual-spatial abilities (Newcombe et al., 2013). Given that gestures are visual-spatial entities and help activate, maintain, and manipulate visual-spatial information (Kita et al., 2017), individual differences in visual-spatial abilities during childhood might affect how much children use gestures and benefit from using gestures for learning. However, there is no direct evidence on how individual differences in verbal and visual-spatial skills relate to children’s gesture use, which begs for future research.

### Individual Differences in Gesture Production in Young Adults

Most of the research on individual differences in gesture production focused on young adults. Studies showed that young adults with lower cognitive capacities used more spontaneous gestures and benefited more from using gestures (e.g., Chu et al., 2014; Gillespie et al., 2014; but see Hostetter and Alibali, 2007), supporting the functionalist accounts (Goldin-Meadow et al., 2001; Marstaller and Burianová, 2013; Kita et al., 2017) and gesture’s role as a compensation tool. The visual-spatial cognitive capacity is related to how much speakers employ gestures during speaking and thinking. People with lower visual and spatial WM capacities, mental rotation skills, and spatial conceptualization abilities (Kita and Davies, 2009) used more gestures compared to high-spatial ability individuals when explaining abstract phrases or social dilemmas (Chu et al., 2014). In a spatial gesture elicitation task, Göksun et al. (2013a) asked young adults to describe how they solved mental rotation problems and found that people with lower spatial abilities (lower mental rotation scores) used more gestures compared to people who had higher scores. However, low‐ and high-spatial ability individuals not only differed in the frequency of gestures but also in the type of gestures they used. People with low-spatial ability used more static gestures depicting objects (i.e., cubes or whole objects), whereas high-spatial ability individuals used more dynamic gestures to express motion, such as rotation or direction or static gestures referring to object pieces (e.g., the bottom part of the L shape). This finding is in line with a previous study showing that although lower‐ and higher-fluid intelligence individuals (as measured by Raven’s matrices) used an equal number of gestures when describing how to solve geometric analogies, people with higher fluid intelligence used more gestures to express motion than people with lower fluid intelligence (Wartenburger et al., 2010; Sassenberg et al., 2011).

Verbal cognitive capacity is another predictor for how and to what extent speakers use gestures (e.g., Baxter et al., 1968; Hostetter and Alibali, 2007, 2011; Nagpal et al., 2011; Smithson and Nicoladis, 2013; Gillespie et al., 2014; for cf. see Frick-Horbury, 2002 and Chu et al., 2014). Young adults with lower verbal abilities such as lower verbal WM capacity, vocabulary size, and semantic fluency (i.e., phonological and lexical retrieval ability) used more gestures during spontaneous speech than individuals with higher verbal abilities (e.g., Hostetter and Alibali, 2007, 2011; Smithson and Nicoladis, 2013; Gillespie et al., 2014; but see Chu et al., 2014). These findings corroborate with bilingual research, showing that bilinguals used more gestures when talking in their L2 compared to L1 or monolinguals (e.g., Gullberg, 1998; Nagpal et al., 2011). Verbal WM also predicted gesture frequency similarly in bilinguals and monolinguals (Smithson and Nicoladis, 2013).

Is there an interaction between verbal and spatial skills in gesture use? Hostetter and Alibali (2007) showed a quadratic relationship between verbal resources and spontaneous gesture use. People with the lowest and highest verbal skills (i.e., phonemic fluency) gestured more than people with average verbal skills when they were retelling a cartoon story and describing how to wrap a package. Moreover, low verbal/high visual-spatial individuals produced the largest number of gestures and used more non-redundant gestures (Vanetti and Allen, 1988; Hostetter and Alibali, 2007, 2011). This might suggest that gestures are more helpful when speakers have spatial information in the non-propositional format in mind but are unable to lexicalize or to encode verbally (e.g., Graham and Heywood, 1975; Krauss and Hadar, 1999).

Young adults also show individual variation in how much they benefit from using gestures during task solving or subsequent memory and learning (e.g., Marstaller and Burianová, 2013). Young adults use many gestures when encoding information that facilitates their subsequent memory and learning, especially for visual and spatial information (e.g., Chu and Kita, 2011; So et al., 2015). However, using gestures is especially beneficial for people with lower cognitive capacity (e.g., Marstaller and Burianová, 2013; Pouw et al., 2016; Galati et al., 2018). People who used gestures when trying to learn new routes had better memory in a subsequent navigation task; however, this was only evident for people with a lower spatial perspective-taking ability (Galati et al., 2018). Moreover, gesturing benefited problem solving under higher cognitive load (e.g., dual-task paradigm; Marstaller and Burianová, 2013) and when internal cognitive resources are taxed or limited (e.g., Pouw et al., 2016).

Individual differences in verbal and visual-spatial skills affect how much young adult speakers use and benefit from producing gestures during speaking and problem-solving. Conforming the *gesture-as-a-compensation-tool* account, speakers employ gestures to compensate for lower verbal and spatial cognitive resources. However, we should be cautious about the generalizability of these findings as to the use of different cognitive measures, and gesture elicitation tasks (e.g., spatial vs. non-spatial abstract) might yield different results. Further research is needed to replicate these conclusions across contexts.

### Individual Differences in Gesture Production in Healthy Aging

Evidence on spontaneous gesture use in healthy aging is minimal. Most of the research compared young and elderly adults and showed that spontaneous gesture production and gesture imitation is impaired in aged populations (e.g., Cohen and Borsoi, 1996; Dimeck et al., 1998; Feyereisen and Havard, 1999). Elderly adults used less representational gestures compared to young adults, whereas overall gesture frequency or the use of non-representational gestures (e.g., beat or conduit gestures) was comparable across two groups (Cohen and Borsoi, 1996; Glosser et al., 1998; Feyereisen and Havard, 1999; Arslan and Göksun, in press; for c.f. see Özer et al., 2017; Schubotz et al., 2019). This might be due to declining visual-spatial cognitive resources in aging. For example, mental imagery declines with aging (e.g., Dror and Kosslyn, 1994; Copeland and Radvansky, 2007; Andersen and Ni, 2008) and, indeed, individual differences in mental imagery, but not spatial WM capacity was associated with how frequently young and elderly individuals used spontaneous gestures, particularly for a spatial address description task (Arslan and Göksun, in press). Elderly individuals were also impaired in designing their multimodal utterances for their addressees (i.e., audience design; Schubotz et al., 2019). When narrating comic cartoons, young adults used fewer gestures when they knew that their addressee also watched the comic cartoon compared to when their addressee did not see the cartoon. However, elderly adults used an equal number of gestures in both cases.

We might expect that declining visual-spatial skills in aging would lead to higher use of gestures by older adults than in younger ones. However, gestures might be used as a compensatory tool only to manage cognitive load when the person has the necessary/intact resources. Most of the studies comparing younger vs. older adults tested individuals who are older than 60 years of age (e.g., Cohen and Borsoi, 1996), and it is unknown whether visual-spatial skills are severely impaired in this age group. Less is also known on the decline in which cognitive abilities in healthy aging leads to age-related impairments in gesture production (but see Arslan and Göksun, in press). It is important to note that this area is open to investigation and future research should study the decline in which cognitive resources lead to impaired gesturing in aging, whether the effects of aging on gesturing is similar for everyone, and which cognitive resources might play a protective role for the decline of gesture production. More research is also needed to examine whether elderly individuals benefit from using gestures as young adults and children do or producing gestures impose an extra cognitive burden to their already limited cognitive resources.

Moreover, we mainly focused on gesture use in healthy aging, yet, the line of research on how people with neurodegenerative disorders use gestures is informative as well (e.g., Cleary et al., 2011; Rousseaux et al., 2012; Klooster et al., 2015; Akhavan et al., 2018; Özer et al., 2019). People with different types of neurodegenerative disorders such as Alzheimer’s disease, primary progressive aphasia, and Parkinson’s disease are natural targets to study gesture in aged populations because the prevalence rates of these diseases consistently increase with age (e.g., Jorm et al., 1987; Brayne et al., 2006). For example, Klooster et al. (2015) showed that the beneficial effects of using and observing gestures on new learning in a Tower of Hanoi paradigm were absent in elderly patients with intact declarative memory, but impaired procedural memory as a consequence of Parkinson’s disease. This suggests that the procedural memory system supports the ability of gestures to drive new learning. Thus, the decline in different memory systems in different neurodegenerative disorders that increase with age might lead to variation in how elderly adults benefit from gestures during learning. Future studies should test the cognitive correlates of impaired gesture use in different neuropsychological groups.

## Individual Differences in Gesture Processing

Listeners are sensitive to speakers’ gestures and benefit from observing these gestures during online language comprehension, encoding, and subsequent memory and learning (Holler et al., 2009; Kelly et al., 2010; Hostetter, 2011; Dargue et al., 2019). The facilitative effects of observing gestures are evidenced across children (e.g., Cook et al., 2008; Austin and Sweller, 2014, 2017; Macoun and Sweller, 2016; Vogt and Kauschke, 2017; Holler et al., 2018; Aussems and Kita, 2019; Dargue and Sweller, 2020; Kartalkanat and Göksun, 2020) and young adults (e.g., Beattie and Shovelton, 1999; Roth, 2001; Holle and Gunter, 2007; Kelly et al., 2008; Hostetter, 2011; Rueckert et al., 2017; Dargue and Sweller, 2020). Research regarding individual differences in how listeners attend to and process speakers’ gestures and how much they benefit from observing gestures during comprehension and learning is quite limited, especially when compared to the literature on individual differences in gesture production (e.g., Post et al., 2013; Wu and Coulson, 2014a,b; Yeo and Tzeng, 2019; Özer and Göksun, 2020). In the next subsections, we review evidence on individual differences in gesture processing and its effects on comprehension and learning in children, young adults, and elderly adults. Then, we discuss several possible cognitive mechanisms that might yield individual differences in gesture processing, suggesting new venues for future research.

### Individual Differences in Gesture Processing in Children

Electrophysiological studies showed that children start to process iconic gestures as semantic entities like words at around 18 months of age (Sheehan et al., 2007). Behaviorally, they start to comprehend iconic gestures representing entities at around 3 years of age (Stanfield et al., 2014) and iconic gestures representing events at around 4 years of age (Glasser et al., 2018). Studies showed that 3-year olds could not integrate speech and gesture, whereas 5-year old and adults did (e.g., Sekine and Kita, 2015; Sekine et al., 2015). Moreover, children starting from 6 years of age integrate speech and gesture in an online fashion comparable to adults (Dick et al., 2012; Sekine et al., in press). Demir-Lira et al. (2018) showed that gesture-speech integration recruits the same neural network as in adults. Yet, this was true only for children who were able to successfully integrate speech and gesture behaviorally. Then, what drives these individual differences in early gesture-speech integration ability?

Gesture-speech integration requires a global developmental shift. The precursors of gesture comprehension and gesture-speech integration are unknown. Gestures are visual-spatial entities and the processing, and the interpretation of gestures requires visual-spatial cognitive resources (e.g., Kelly and Goldsmith, 2004). Children with lower visual-spatial skills might have difficulty in processing and comprehending gestures compared to children with higher visual-spatial skills. The global development of executive attention and general WM capacity, on the other hand, might play a role in gesture-speech integration. For example, children with lower overall WM capacity might have difficulty in maintaining and integrating two different kinds of information simultaneously, especially in offline integration tasks (e.g., Demir-Lira et al., 2018). Cognitive predictors of individual differences in children’s gesture comprehension and gesture-speech integration abilities require further attention.

What about individual differences in the beneficial effects of observing gestures for subsequent learning? Not all children benefit from visual aids such as diagrammatical illustrations when learning math (e.g., (Cooper et al., 2017). Indeed, observing gestures does not assist all children’s comprehension of narratives or learning new skills (e.g., Church et al., 2004; van Wermeskerken et al., 2016; Yeo and Tzeng, 2019; Bohn et al., 2020; Kartalkanat and Göksun, 2020). Kartalkanat and Göksun (2020) found a positive relationship with verbal skills and the beneficial effects of observing gestures, preschoolers with higher expressive language ability benefited more from observing iconic gestures in the encoding of spatial events. Bohn et al. (2020) found that children benefited from observing gestures when learning novel skills (e.g., how to open a novel apparatus) as they became older. On the other hand, Demir et al. (2014) showed that children with pre‐ and perinatal unilateral brain injury (BI) and had difficulty in narrative production benefited more from observing gestures when retelling narratives compared to TD children (Demir et al., 2014). Moreover, children with specific language impairment (SLI) benefited more from observing gestures compared to TD and used the same gestures they observed when retelling the inferred meaning of the spoken messages (Kirk et al., 2011). The contradictory findings regarding the relation between verbal abilities and the beneficial effects of observing gestures between children with language impairments and children with intact language abilities pose a challenge. We might expect TD children with lower verbal abilities to benefit more from observing gestures as predicted by *gesture-as-a-compensation-tool* account; however, young children’s limited verbal resources might be already consumed with processing speech, leaving few resources to process and benefit from external visual cues (i.e., gestures; Kalyuga, 2007). Again, gestures might help children manage cognitive load when they have fully developed verbal abilities. However, children with language impairments might employ gestures to compensate for their already-impaired spoken language abilities.

Individual differences in verbal (e.g., digit span task; Kartalkanat and Göksun, 2020), visual (e.g., visual patterns task; van Wermeskerken et al., 2016), or general WM capacity (e.g., operation span task; Yeo and Tzeng, 2019) did not predict how much children benefited from observing gestures for learning. There was hardly any variance in WM capacity in most of these studies (e.g., van Wermeskerken et al., 2016). This might obscure the otherwise possible effects of different WM capacities on the values of observing gestures in children. Additionally, how general spatial skills (e.g., mental rotation and mental imagery) relate to how much children benefit from observing gestures needs to be investigated in future research.

### Individual Differences in Gesture Processing in Young Adults

Young adults also differ in how they process spontaneous co-speech gestures. Processing gestures require visual, spatial, and motoric cognitive resources (e.g., Kelly and Goldsmith, 2004; Wu and Coulson, 2014a). We expect people with higher visual-spatial abilities to process and comprehend gestures better. Indeed, Wu and Coulson (2014a) found that people with higher spatial WM (but not verbal WM) were better at processing co-speech gestures as they were more sensitive to speech-gesture mismatches (i.e., high-spatial individuals were affected more negatively when gesture and speech expressed incongruent information). Moreover, people who have larger spans for retaining and manipulating bodily configurations (i.e., motor movement span task assessing individuals’ ability to retain body-centric motor information) comprehended gestures better (Wu and Coulson, 2014b). In a recent study, we asked how visual-spatial vs. verbal WM capacity relates to processing concurrent visual (i.e., gesture) and verbal (i.e., speech) information in a mismatch paradigm used initially by Kelly and colleagues in 2011 (Özer and Göksun, 2020). We demonstrated that listeners showed differential sensitivity in processing concurrent gestural vs. spoken information. Although gesture-speech mismatches hindered overall comprehension, how listeners got affected by mismatches in different modalities (gesture vs. speech mismatches) was dependent on the listeners’ cognitive dispositions on visual-spatial vs. verbal resources. Observing mismatching visual information (i.e., gesture) imposes an additional visual-spatial cognitive load and people with higher spatial abilities were better at maintaining and processing two different and mismatching visual information due to their higher capacity. As a result, these individuals perform better when gestures expressed mismatching information compared to people with lower spatial abilities. People with higher verbal abilities, on the other hand, had better performance when speech expressed mismatching information compared to people with lower verbal abilities. These findings suggest that visual-spatial cognitive resources are critical for gesture processing and observing mismatching gestures increase visual-spatial cognitive load (e.g., Kelly and Goldsmith, 2004; Hostetter et al., 2018). Processing mismatching information in visual modality would be less demanding for people with larger visual-spatial cognitive resources.

What about individual differences in how much listeners benefit from observing gestures? Earlier studies are limited in suggesting how listeners integrate visual information with speech and use gestures to encode information either for online language comprehension or for subsequent learning. Research on how learners benefit from different multimedia materials (visual vs. verbal representations) might give us insight in this matter (Ausburn and Ausburn, 1978; Kirby et al., 1988; Koć-Januchta et al., 2017; Kiat and Belli, 2018; but see Kirschner, 2017). Individuals show variation in how they benefit from visual vs. verbal information (Kirby et al., 1988; Riding et al., 1995; Kozhevnikov et al., 2002; Mendelson and Thorson, 2004; Meneghetti et al., 2014; Alfred and Kraemer, 2017). For example, learners show variation in how they fixated to text vs. pictures when learning from multimedia resources (Koć-Januchta et al., 2017) and students with higher spatial abilities benefited more from the presence of 3D models when learning cell biology compared to students with lower spatial abilities (Huk, 2006). This suggests that listeners’ cognitive dispositions might be related to how much they benefit from observing gestures vs. hearing speech. A very recent study directly tested how different WM capacities related to how much young adults benefited from observing gestures (Aldugom et al., 2020). Undergraduate students with higher visual WM capacity (i.e., visual patterns task) benefited more from observing gestures during math learning whereas verbal (i.e., sentence span task) and motoric (i.e., movement span task, Wu and Coulson, 2014b) WM capacities did not predict the beneficial effects of observing gestures (Aldugom et al., 2020). Although it is well-established in the literature that gestures facilitate listeners’ comprehension and learning (see Özyürek, 2014 for review), evidence suggests that this is not a monolithic process. It is also possible that observing gestures do not always facilitate comprehension and learning. For example, observing gestures hurt learning phonetic distinctions at the syllable level within a word for English-speaking adults learning vowel length contrasts in Japanese (Kelly et al., 2014). However, as in the case with children (Kartalkanat and Göksun, 2020), learners’ level of second-language proficiency might play a role for benefitting from gestures, which is another cognitive resource to be examined. Future studies should investigate the cognitive precursors of individual differences in the beneficial effects of observing gestures across different learning contexts (e.g., spatial vs. non-spatial) and different stages of language processing (e.g., phonological vs. semantic; Kelly et al., 2014).

### Individual Differences in Gesture Processing in Healthy Aging

Few studies examined how elderly individuals process gestures and benefit from observing gestures (e.g., Thompson, 1995; Ska and Croisile, 1998; Montepare et al., 1999; Thompson and Guzman, 1999; Cocks et al., 2011). Elderly individuals are impaired in their comprehension of pantomimes and emotional gestures compared to young individuals (Ska and Croisile, 1998; Montepare et al., 1999). Moreover, elderly adults are impaired in integrating speech and gesture compared to young adults (Cocks et al., 2011). However, they performed equally when two cues are presented in isolation, suggesting that they might be impaired in gesture-speech integration with a preserved ability to process gestures. Indeed, elderly adults mostly relied on visible speech and did not benefit from observing gestures when recalling sentences (Thompson, 1995).

Although young adults benefited from visual aids (i.e., visible speech and gestures) under challenging listening conditions (i.e., dichotic shadowing task), older adults did not (Thompson and Guzman, 1999). The differences in the effects of observing gestures between younger and older adults might be related to the declining cognitive abilities associated with aging, mainly due to WM capacity as WM is required to maintain and manipulate different information. However, this has not been addressed directly.

Previous research suggests that elderly adults have difficulty in integrating visual (i.e., gesture) and verbal (i.e., speech) information compared to younger adults. It might be due to a decline in global cognitive skills such as executive attention and general WM capacity. Yet, it has not been directly tested. Future studies should compare younger vs. older adults with several cognitive measures to understand the cognitive architecture behind impaired gesture processing and gesture-speech integration in healthy aging.

## Conclusion and Future Directions

Speakers use gestures as they speak and think, and listeners, in turn, are sensitive to speakers’ gestures. Gestures (both using by speakers and observing by listeners) have beneficial effects on language comprehension, problem-solving, encoding, and subsequent learning. Studies, to date, mostly focused on the role of different external factors (e.g., speech content and communicative context) on gestural behavior to answer *when* we use and benefit from gestures. However, it is also essential to ask *who* uses and benefits from gestures for *which* purposes. Research on the cognitive precursors of these individual differences in gesture use and processing has just started to emerge. Examining individual differences in gesture use and processing will help us uncover the cognitive architecture behind these processes and inform gesture research that is based on group data. The accounts that explain how and why gestures are employed should integrate individual differences research to have a full picture of when, why, and for whom gestures exhibit their supposed roles. This line of research is also informative for the development of educational programs incorporating the use of gestures by learners or teachers. The instructional programs should be tailored according to the cognitive dispositions and needs of the learners for optimal learning outcomes.

Most of the research on individual differences in the gesture literature examined gesture production in young adults. Studies on gesture use in children and elderly adults focused on group comparisons (i.e., comparing children at different ages, children vs. adults, younger vs. older adults, and clinical vs. non-clinical groups). Moreover, individual differences in gesture processing are limited compared to the production literature. In the current review paper, we (1) combined two lines of research: using gestures and observing gestures and (2) discussed the possible cognitive precursors of gesture use and processing in different age groups. We also highlighted the functions of producing and seeing gestures regarding their compensatory roles in speaking and thinking.

Gestures provide an alternative expression channel and assist speakers and listeners communicate (e.g., Alibali et al., 2001; Hostetter, 2011). Gestures also decrease speakers’ and listeners’ cognitive load by aiding them to activate, maintain, and manipulate visual-spatial information (e.g., Kita et al., 2017; Novack and Goldin-Meadow, 2017). Gestures help people manage cognitive load and are used as a compensatory tool. Listeners’ and speakers’ cognitive dispositions interact with this compensatory role of gestures, leading to individual differences in how much people benefit from using and seeing gestures for speaking, comprehension, task solving, and learning. As the *gesture-as-a-compensation-tool* account would argue, children and adults with lower cognitive resources use and benefit from using gestures more to manage cognitive load compared to people with higher cognitive resources (e.g., Church et al., 2000; Göksun et al., 2010; Marstaller and Burianová, 2013; Austin and Sweller, 2014; Chu et al., 2014; Gillespie et al., 2014; Galati et al., 2018). However, we suggest that gestures do not replace the impaired cognitive abilities; instead, gestures help manage cognitive load when cognitive resources are intact. Gestures might not compensate for already-impaired cognitive abilities. For example, people with aphasia use more gestures to compensate for impaired speech, but only when they have the intact conceptual knowledge of what they express (e.g., Göksun et al., 2013b, 2015). In a similar vein, there is a decrease in gesture production in healthy aging that might be due to impaired visual-spatial abilities such as mental imagery (e.g., Cohen and Borsoi, 1996; Arslan and Göksun, in press). There is also evidence of individual differences in gesture processing. Processing and comprehending gestures require visual-spatial cognitive resources (e.g., Kelly and Goldsmith, 2004; Hostetter et al., 2018). People with higher visual-spatial skills (or older children compared to younger children) process gestures better compared to people with lower visual-spatial skills (e.g., Wu and Coulson, 2014a,b; Özer and Göksun, 2020). In line with the *gesture-as-a-compensation-tool* account, we expect that people with lower cognitive resources (especially visual-spatial) would benefit more from observing external visual cues (i.e., gestures, but see Aldugom et al., 2020). However, research on how visual-spatial abilities relate to how much listeners benefit from observing gestures is inconclusive and begs for further investigation.

Although group-comparison studies are informative, future work should address within-group variation more, especially in children and in elderly adults. How different cognitive skills are associated with gesture production and processing should be tested directly across different conditions. The employment of different cognitive measures, gesture elicitation tasks, and learning contexts might yield different results, and these should be incorporated to have a full picture of whom for and when gestures are helpful. For example, the relationship between visual-spatial abilities and how frequently speakers use gestures and how much they benefit from using and observing gestures depend on the content of the information to be communicated or learned (Lausberg and Kita, 2003; Hostetter and Alibali, 2007; Chu et al., 2014; Arslan and Göksun, in press). The role of visual-spatial abilities in gesture use and processing might be more pronounced in spatial speech compared to non-spatial speech (e.g., Alibali, 2005; Arslan and Göksun, in press). Future work should also investigate how internal sources of variation (e.g., individual differences in several abilities) interact with external sources of variation (e.g., speech content and task difficulty).

One area open for future investigation is the cognitive predictors of gesture processing; that is, how listeners attend, process, and benefit from observing gestures. Studies on how visual, verbal, and motoric WM capacities are linked to individuals’ processing of concurrent gesture vs. speech employed mismatch paradigms (Wu and Coulson, 2014a,b; Özer and Göksun, 2020). However, gesture mismatches are rare in natural communication, and we should investigate how different cognitive abilities relate to gesture processing in more ecologically valid paradigms. It is also unknown whether there are any individual differences in visual attention to gestures. Gestures are visual articulators and subject to visual processing. Although earlier research found that gestures can be processed peripherally and do not require direct visual attention (e.g., Gullberg and Holmqvist, 1999, 2006; Gullberg and Kita, 2009), recent evidence suggests that several factors might modulate how listeners allocate overt visual attention to gestures such as the comprehensibility of speech, and the native/non-native status of the listener (e.g., Drijvers et al., 2019). Future studies should address whether people with different visual-spatial vs. verbal abilities show differential overt visual attention to gestures and how this relates to individual differences in gesture processing (Wakefield et al., 2018). Above attending to and processing gestures, very little is known on whether and how individuals benefit from observing gestures during online language comprehension and learning across different learning contexts (Aldugom et al., 2020). We currently investigate how visual-spatial skills relate to how much listeners benefit from observing gestures when comprehending spatial relations between objects.

All studies reviewed above tested individual differences behaviorally. Electrophysiological and neuroimaging studies investigate the neural architecture of gesture use and processing (e.g., Kelly et al., 2004; Wu and Coulson, 2005; Willems et al., 2009). We might observe individual differences in neural data, that is, otherwise non-observable behaviorally (e.g., Demir-Lira et al., 2018). Future work should examine individual differences in the recruitment of different neural networks when using and observing gestures and how these differences in neural data relate to behavioral performance after considering individuals’ cognitive skills.

The current review only focused on how individual differences in cognitive skills (mostly verbal and visual-spatial skills) relate to gesture use and processing. However, individual differences in other domains might also affect how people employ gestures. Individual differences in other domains such as personality (Hostetter and Potthoff, 2012) and other aspects of cognitive and perceptual skills such as selective attention, auditory processing, and the speed of multisensory processing should be tested (e.g., Schmalenbach et al., 2017). Moreover, it is also important to study the relation between gesture production and processing. How individual differences in spontaneous gesture use predict how people attend to and benefit from observing gestures or vice versa are unknown (Wakefield et al., 2013). Gesture processing might be affected by to what extent people themselves use gestures, and future studies should address the production-perception cycle and the mechanisms behind it.

In sum, gesture use and processing are not monolithic processes and show individual variation. Speakers and listeners can use gestures as a compensation tool during communication and thinking that interact with individuals’ cognitive dispositions.

## Author Contributions

DÖ and TG conceived of the presented idea. DÖ drafted the manuscript. TG revised the manuscript critically for important intellectual content. Both the authors contributed to the article and approved the submitted version.

### Conflict of Interest

The authors declare that the research was conducted in the absence of any commercial or financial relationships that could be construed as a potential conflict of interest.

The reviewer declared a shared collaboration in a society with one of the authors TG at time of review.
